# Bioinspired morphology and task curricula for learning locomotion in bipedal muscle-actuated systems

**DOI:** 10.1038/s44172-025-00443-0

**Published:** 2025-06-20

**Authors:** Nadine Badie, Firas Al-Hafez, Pierre Schumacher, Daniel F. B. Haeufle, Jan Peters, Syn Schmitt

**Affiliations:** 1 Institute for Modelling and Simulation of Biomechanical Systems, University of Stuttgart, Nobelstraße 15, Stuttgart, 70569 Germany; 2 Institute for Intelligent Autonomous Systems, TU Darmstadt, Hochschulstraße 10, Darmstadt, 64289 Germany; 3 Max Planck Institute for Intelligent Systems, Max-Planck-Ring 4, Tübingen, 72076 Germany; 4 Hertie-Institute for Clinical Brain Research, University of Tübingen, Otfried-Müller-Str. 27, Tübingen, 72076 Germany; 5 Center for Bionic Intelligence Tübingen-Stuttgart (BITS), Universities of Stuttgart and Tübingen, Nobelstraße 15, Stuttgart, 70569 Germany

**Keywords:** Developmental biology, Biomimetics, Mathematics and computing, Engineering

## Abstract

Humans master complex motor skills such as walking and running through a sophisticated blend of learning and adaptation. Replicating this level of skill acquisition with traditional Reinforcement Learning (RL) methods in musculoskeletal humanoid systems is challenging due to intricate control dynamics and over-actuation. Inspired by human developmental learning, here we address these challenges, with a double curriculum approach: a three-stage task curriculum (balance, walk, run) and an up to three-stage morphology curriculum (4 year-old, 12 year-old, adult), mimicking physical growth. This combined approach enables the agent to efficiently learn robust gaits that are adaptable to varying velocities and perturbations. Extensive analysis and ablation studies demonstrate that our method outperforms state-of-the-art exploration techniques for musculoskeletal systems. Our approach is agnostic to the underlying RL algorithm and does not require reward tuning, demonstrations, or specific muscular architecture information, marking a notable advancement in the field.

## Abstract (web summary)

Achieving locomotion in musculoskeletal humanoid systems is a challenge for reinforcement learning. Badie and colleagues propose a curriculum combining physical growth and skill progression to develop gaits adaptable to varying speeds and terrains.

## Introduction

Humans demonstrate an unmatched learning ability, dexterity and versatility in locomotion compared to robotic systems^1^. Even with advanced machine learning techniques that arguably mimic biological strategies like RL^2^, these human attributes remain unparalleled, especially in muscle-actuated systems.

Acquiring locomotion in humans is complex, driven by both morphological and motor skill development. Morphology pertains to the physical structures and form of the body, while motor skills involve the tasks that individuals learn and perform. Most motor skills are acquired during the formative phase of physical development^3^. Infants begin with basic movements and progressively advance to more sophisticated actions like walking and running as their bodies mature. As individuals transition from childhood to early adulthood, they continue to refine their locomotion abilities, gaining increased proficiency and versatility in movement.

Replicating human motor skills in artificial systems to advance technologies is an intricate challenge. Bipedal gaits, particularly, pose major obstacles, with RL struggling to efficiently navigate the complex state space^4,5^. This difficulty is compounded in multi-task learning environments, where balancing competing demands, such as adapting to varying walking speeds, increases the observation and transition spaces, often resulting in slow convergence, local optima, and instability^6,7^. When considering musculoskeletal systems, these challenges are amplified. Unlike torque-actuated robots, muscles can only contract, necessitating a complex agonist-antagonist setup and coordinated control to prevent force cancellation. Additionally, short muscle twitches often fail to produce adequate joint motions due to chemical low-pass filter properties^8^. This over-actuated system, with more muscles than degrees of freedom (DOF), and intricate dynamics, presents a challenging control problem. To manage this difficulty, several strategies have been developed to enhance exploration and accelerate learning. One approach is imitation learning, which uses human demonstrations to guide robotic controllers toward typical human movement patterns^9^. While it has shown success in various studies^10,11^, it requires extensive data and may not be suitable for all environments. Alternatively, demonstration-free techniques like noise-based exploration^12^ or low-level representation learning^13–15^ offer another solution. Some approaches promote exploration by adding zero-mean noise^6^, while others focus on stabilizing exploration over time with temporally correlated or colored noise^12,16^. Count-based exploration encourages less-visited states, and parametric noise improves efficiency compared to action space noise^6^. Deep exploration strategies, with multi-head structures in value functions, diversify action selection^17^. We refer to Geiß et al.^11^ for further discussion regarding exploration in muscle-driven systems. One particular approach, differential extrinsic plasticity (DEP) leverages self-organization to enhance state-space exploration by maximizing correlations between muscle length velocities^18,19^. Providing more effective exploration, it has been shown to surpass traditional noise-based methods in learning locomotion^19,20^. Despite these advancements, DEP-RL and similar methods often seem to lack the structured progression necessary for mastering multiple complex tasks. Curriculum learning addresses this by systematically increasing task complexity in a manner inspired by human learning phases^21^. Weng et al.^22^ used a nature-inspired task curriculum, beginning with simpler behaviors like standing and stepping, before progressively refining gait in muscle-actuated systems. However, achieving close-to-natural gaits in 2D required extensive neuromechanically-inspired reward tuning.

**Fig. 1:**
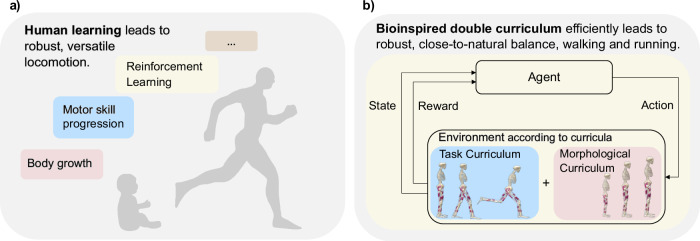
Bioinspired double curriculum approach for efficiently learning robust standing, walking, and running. **a** Humans acquire versatile motor control through Reinforcement Learning (RL), using trial and error to optimize actions based on rewards, while simultaneously undergoing morphological and motor skill development. **b** Drawing from human development, our bioinspired double curriculum approach tackles RL exploration challenges in musculoskeletal models by integrating two curricula: a morphological curriculum based on growing body morphologies and a task curriculum, progressing from standing to walking and eventually running.

While current methods increase the complexity of algorithm or reward design, our approach shifts the focus to another dimension: morphology. Although previous work has applied morphological development to simple tasks using optimal control^23^, its application to complex, muscle-actuated locomotion in RL remains unexplored. We address this gap by introducing a bioinspired double curriculum framework (Fig. 1), leveraging embodied intelligence— the inherent relationship between physical structure and agent learning. In our framework, embodied intelligence is expressed through morphological intelligence, where the musculoskeletal model itself, especially at different developmental stages, enhances exploration and learning. First, we evaluate this by scaling down the adult musculoskeletal model into younger morphologies and analyzing their kinematic workspace coverage to demonstrate improved exploration. Second, we combine the growth component with motor skill progression inspired by humans, designing a bioinspired double curriculum that efficiently enables learning of upright standing, walking, and running within a unified policy. Through ablation studies and benchmarking, we demonstrate the reliability of our framework and its superiority over existing approaches like DEP-RL. Third, we conduct a systematic robustness assessment of the learned gaits, revealing their adaptability to perturbations and varying velocities. In summary, by utilizing embodied intelligence to enhance the algorithm’s capabilities, our framework achieves state-of-the-art locomotion performance with a standard two-layer architecture, eliminating the need for reward shaping, intricate exploration methods, or demonstrations.

## Results

### Conceptual design of bioinspired curricula

Our objective is to train an agent capable of proficiently executing standing, walking, and running tasks, using RL. To overcome exploration challenges, we employ a bioinspired double curriculum approach. Curricula break down complex tasks into simpler stages, facilitating learning through guided progression^21^. Knowledge gained rapidly from simple tasks can be utilized to minimize the exploration required for more complex tasks^24^. Two widely used types of curricula are environment-based and task-based curricula. While previous studies have used them separately, our work combines them for musculoskeletal systems learning a wide range of movements.

#### Morphological curriculum

Our bioinspired double curriculum approach innovates in environment-based curricula by modifying the musculoskeletal model itself rather than just altering the terrain or assistive forces^25–27^. We hypothesize that this bioinspired growth approach directly enhances exploration rather than merely simplifying the environment.

To assess the impact of morphology on exploration behavior, we scale down the adult model to approximate a 4 year-old. The 4 year-old morphology was chosen because this developmental stage is characterized by motor control refinement and rhythmic coordination of basic skills^3^. Furthermore, rather than the development of the central nervous system, the changes in walking that take place beyond that age can be linked to changes in limb length, which is the main emphasis of this work^28^. We create two versions of 4 year-olds by scaling segment lengths, masses, moments of inertia, as well as muscle lengths and forces. The first, Onto4y, follows human ontogenetic development, scaling segments proportionally based on biological growth patterns. The second, Uni4y, applies uniform scaling across all segments. While both variants maintain the same total mass, they differ in segment proportions, mass distribution, and consequently muscle lengths and forces. “Environment” in section Methods provides further details on the selected age and the scaling procedure applied to all relevant biomechanical parameters mentioned^23^.

**Fig. 2:**
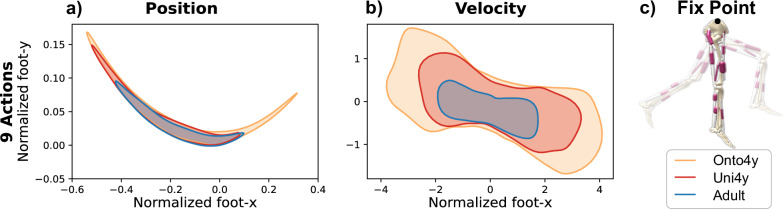
Younger morphologies, particularly the ontogenetically scaled (Onto4y) followed by the uniformly scaled (Uni4y) 4 year-olds, exhibit enhanced exploration, quantified by an expanded kinematic workspace with greater position and velocity coverage. Foot trajectories and velocities were captured across 50 episodes of 1000 steps each (Δ*t* = 10 *s*) during pure exploration. **a** Dimensionless position and (**b**) velocity data, normalized according to the typical biomechanical approach of Hof^60^, are presented. **c** Schematic representation of the experiment, in which the pelvis of each morphology was constrained and one leg was perturbed with white noise.

To illustrate how the 4 year-old morphology enhances exploration compared to the adult morphology, we designed a toy experiment using a simplified version of these musculoskeletal models. Unlike the models used in the remaining results, the version for the toy experiment fixed the pelvis, excluded the adductor and abductor muscles in both legs, restricting leg movement to 2D, and immobilized one leg. Pure white noise was applied to the muscles of the free leg, allowing us to observe the resulting exploratory behavior. Exploration was quantified by tracking the free foot’s center of mass trajectory and velocity over time. These reflect both the spatial range covered by the foot’s center of mass and the velocity at which the foot moves through this space. As shown in Fig. 2, the Onto4y covers the largest space and velocity range, indicating the highest level of exploration, followed by Uni4y and the adult model. This increased exploration can be attributed to the uneven mass distribution of Onto4y, which has a heavier torso but the lightest legs, resulting in lower rotational inertia and thus enabling more extensive and faster leg movements. To test the robustness of the exploration in systems with greater degrees of over-actuation, we conducted this toy experiment with an action space multiplier. See Supplementary Materials 1.1 with the results displayed in Supplementary Fig. 1.

Building on these findings, for our morphological curriculum design, we initially started with a 4 year-old, transitioning through a 12 year-old, and culminating in an adult morphology. The 12 year-old morphology was chosen as a pre-growth spurt phase, avoiding the disproportionate bone mass increase at around age 14^29^. However, as detailed in “Schedule ablation”  in section Results, no discernible efficiency advantages were associated with the inclusion of the 12 year-old. Prioritizing efficiency, we excluded the 12 year-old model from the main curricula.

#### Task curriculum

For the task-based curriculum, we draw inspiration from how humans naturally learn movement, similar to Weng et al.^22^. Humans progress from learning to balance and stand to taking steps, walking, and eventually running. Mimicking this progression, we create a three-phase curriculum:

- Balancing phase: The agent starts by mastering balance at 0 ms-1.
- Walking phase: The agent continues tuning balance while also learning to walk with a velocity of 1.2 ms-1 for the adult model. This velocity was chosen as it closely aligns with the average energetically optimal human walking speed^30^, which has been established in the literature as a reference for efficient human gait^11,20,22^. The choice of a specific velocity is consistent with young children preferably learning one speed at first and progressing to more velocities as they gain experience^31^.
- Running phase: The agent progresses to mastering balance, walking, and running at different speeds up to 3.4 ms-1. This maximum represents a natural outdoor running speed observed in participants running 4 km^32^, and it approximates the highest velocity achieved by the model when optimizing for maximum forward velocity in the reward across three different seeds.

To ensure fairness, the target velocities are scaled to each musculoskeletal model to ensure smaller models learn movements at appropriate speeds, preserving dynamic similarity^33^.

#### Sequencing of curricula

One of the ongoing challenges in curriculum learning is determining the optimal sequencing of tasks. Currently, there is no universal solution for effective sequencing, making it an active area of research^24^. In our study, we draw inspiration from biological development. Early life is characterized by rapid growth and learning, laying the groundwork for future skills. Building on the concept of early-stage development, our study compares three strategies against a baseline non-curriculum:

- Non-curriculum (NonCurr): The agent directly learns the running phase without any predefined curriculum.
- Task curriculum (AdultCurr): The agent follows the task curriculum using the adult morphology throughout. It starts in the balance phase, transitioning to the walking phase at 2 × 10^6^ environment steps and then to the running phase at 4 × 10^6^ steps.
- Ontogenetic double curriculum (OntoCurr) and uniform double curriculum (UniCurr): These curricula combine both morphological and task curricula. Starting with the respective 4 year-old model (Onto4y or Uni4y), the agent learns to balance until 2 × 10^6^ environment steps, transitions to the walking phase until 4 × 10^6^ steps, and then progresses to the running phase until 10 × 10^6^ steps. Subsequently, the agent switches to the adult morphology and continues learning the running phase.

### Benchmarking locomotion learning

With the curricula designed to mitigate the exploration challenges inherent in RL, we apply Maximum a Posteriori Policy Optimisation (MPO)^34^ to our different strategies to efficiently learn robust gaits. Details on our experimental design can be found in the section “Methods”.

**Fig. 3:**
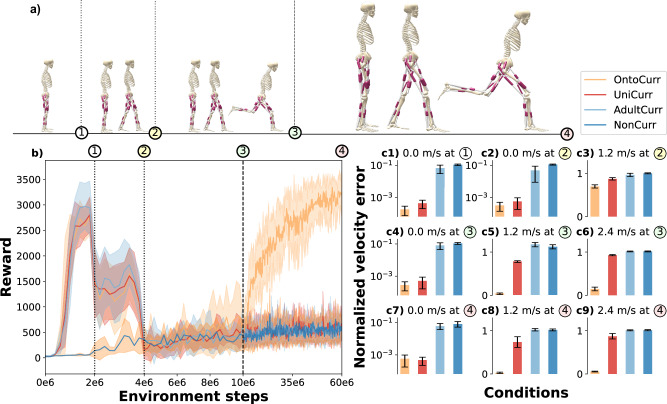
Ontogenetic morphological curriculum combined with task curriculum (OntoCurr) successfully learns all target velocities. OntoCurr outperforms the baseline without curriculum (NonCurr) and two alternative curricula: the task curriculum (AdultCurr) and the uniform morphological and task curriculum (UniCurr). **a** shows a schematic representation of the different phases of the bioinspired double curriculum, combining both task and morphology curricula. **b** demonstrates the means and standard deviations of rewards for ten seeds for all four conditions. Dotted lines denote task transitions, while the dashed line marks the transition to the adult morphology, relevant only for UniCurr and OntoCurr. (**c1-c9**) illustrate the velocity errors with standard deviations represented by error bars at three transition checkpoints (2 × 10^6^, 4 ×  10^6^, 10 × 10^6^) and the final checkpoint (6 × 10^7^) across ten rollouts in each of the ten seeds (*n* = 100) for a sample of target velocities: 0.0 ms-1 (balance), 1.2 ms-1 (walking), and 2.4 ms-1 (running). For balance, velocity errors are normalized by episode length to account for early termination. For walking and running, errors are normalized by target velocity to ensure fair comparison across musculoskeletal models.

Figure 3b depicts the mean and standard deviation of rewards across the four experimental conditions, each with ten seeds. Vertical dotted and dashed lines denote key phases of the curricula, described above. The NonCurr condition exhibits consistently low rewards throughout the training, showing no discernible trend. In contrast, AdultCurr, UniCurr, and OntoCurr display a strong initial increase in rewards during the balance phase, with AdultCurr achieving the highest peak. Upon transitioning to the subsequent phase, all three strategies experience a sharp decline to approximately half of their peak rewards. At the next transition point, rewards plummet sharply for the three strategies. AdultCurr and UniCurr remain low, comparable to NonCurr. However, OntoCurr shows a modest increase during the running phase with the Onto4y morphology. Upon transitioning to the adult morphology, OntoCurr initially experiences a decrease in rewards, but immediately recovers and continues to rise, converging towards a maximum around 3000. We train all strategies for 6 × 10^7^ environment steps to ensure that none of the initially unsuccessful strategies learn substantially later.

In addition, we assess the performance by comparing the target velocities to the mean velocities achieved at the transition and final checkpoints across ten rollouts for all seeds. We evaluate three distinct velocities: 0.0 ms-1 for balance, 1.2 ms-1 for walking, and 2.4 ms-1 for running. For balance velocity errors are adjusted according to episode length to account for premature terminations. For walking and running, errors are scaled by the target velocity to facilitate a fair comparison between different musculoskeletal models. Overall, the velocity errors for balance are notably consistent across the various checkpoints (Fig. 3c1, c2, c4, c7). At the first checkpoint, OntoCurr achieves the smallest error, followed by UniCurr, AdultCurr, and NonCurr. This trend is maintained at the second and third checkpoints. However, by the final checkpoint, UniCurr slightly outperforms OntoCurr, though both exhibit substantially lower errors compared to AdultCurr and NonCurr. The walking task is evaluated at the second checkpoint, as the double curricula initially focus on balancing until the first checkpoint (Fig. 3c3, c5, c8). OntoCurr once again achieves the lowest error, with UniCurr exhibiting a slightly higher error, and both AdultCurr and NonCurr displaying the highest errors. This pattern continues through the third and fourth checkpoints, with OntoCurr’s error strongly decreasing. Running is first evaluated at the third checkpoint due to the progression of the double curricula (Fig. 3c6, c9). At this and the final checkpoint, OntoCurr demonstrates a considerably lower error compared to the other conditions.

#### Schedule ablation

**Fig. 4:**
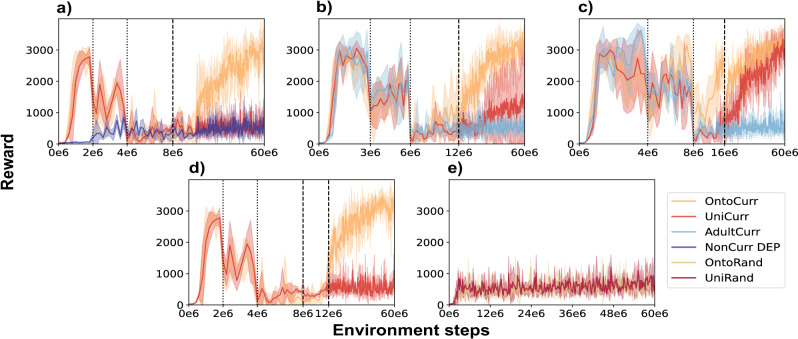
Ablation experiments demonstrate the general applicability and minimal impact of curriculum schedules. We compare the ontogenetic morphological and task curriculum (OntoCurr), the uniform morphological and task curriculum (UniCurr), only the task curriculum (AdultCurr), and random morphology schedules with uniform (UniRand) and ontogenetic (OntoRand) scaling. We test five curriculum schedules, differentiated by curriculum and morphology switch points (dotted and dashed lines, respectively), and present the corresponding means and standard deviations of rewards. **a** Schedule 2-4-8 includes 2 × 10^6^ curricula increments and is complemented by a non-curriculum baseline employing state-of-the-art DEP-RL^19^. To maintain clarity, the AdultCurr condition, identical to the schedule in the main results, is not presented. **b** Schedule 3-6-12 assigns 3 × 10^6^ step increments. **c** Schedule 4-8-16 gives 4 × 10^6^ step increments. **d** Schedule 2-4-8-12 builds on (**a**) allocating steps 8 × 10^6^ to 12 × 10^6^ to the corresponding 12 year-old morphology before switching to the adult morphology and is thus only presented for double curricula strategies. **e** The fifth schedule abandons both morphology and task curricula, opting for random switches in both morphology and velocity.

We experiment with various curriculum schedules (Fig. 4), demonstrating the generality and consistency of our results, as well as the indifference of curriculum variations, particularly evident in the highly successful OntoCurr. Since the initial phases are similar to the main results (Fig. 3b) across all schedules that include a task curriculum, we focus on the period after the transition to running, unless there are considerable dissimilarities. The ablations with three seeds each are:

- Schedule 2-4-8: First, we shorten the running phase before the transition to the adult morphology to 8 × 10^6^ instead of 10 × 10^6^. We consider OntoCurr and UniCurr, as the AdultCurr version is identical due to the absence of a transition to adult morphology. As displayed in Fig. 4a only the OntoCurr strategy increases at 10 × 10^6^ and converges to rewards around 3000, while UniCurr rewards remain low.
- Schedule 3-6-12: This schedule extends the duration of all phases using increments of 3 × 10^6^ instead of 2 × 10^6^. Extending these phases aims to improve balance and walking learning for all strategies and allow younger models more time to adapt to the running phase. At the final transition, while the AdultCurr rewards remain spiky and low, the OntoCurr rewards increase and converge (Fig. 4b). Differently from other schedules, the UniCurr starts rising at around 3 × 10^7^, but reaches a lower mean and a high standard deviation compared to OntoCurr.
- Schedule 4-8-16: We extend the schedule duration to increments of 4 × 10^6^, resulting in the final transition to the adult stage at 16 × 10^6^ for the combined curricula. During the 4 year-old running phase, OntoCurr rewards spike and converge, while UniCurr rewards start increasing modestly (Fig. 4c). Although rewards initially drop at the morphology transition, both OntoCurr and UniCurr quickly recover and converge to similar values. Differently, AdultCurr rewards remain low during the running phase.
- Schedule 2-4-8-12: This schedule includes a 12 year-old morphology and extends Schedule 2-4-8. At 8 × 10^6^, the agent transitions from the 4 year-old to the 12 year-old morphology and continues learning in the running phase until 12 × 10^6^, then transitions to the adult morphology. As shown in Fig. 4d, OntoCurr starts increasing rewards during the 12 year-old morphology phase and converges towards higher values after transitioning to the adult morphology, while UniCurr remains consistently low. There was no clear improvement in efficiency and final rewards compared to the 2-4-8 or 2-4-10 schedules. To simplify our approach, we excluded the extended morphology schedule with the 12 year-old model from our current approach.
- Random morphology: In this non-bioinspired setup, the agent randomly alternates between the 4 year-old and adult morphologies throughout the entire training process, without adhering to a task curriculum. Thus, it only learns the running phase with velocities sampled randomly. We test this artificial approach based on its application in previous work^35^. Both the ontogenetic (OntoRand) and uniform (UniRand) versions result in spiky low rewards (Fig. 4e). More variants of the random morphology experiments can be found in Supplementary Materials 1.2 and Supplementary Fig. 2.

#### Comparison to DEP-RL

To contextualize our findings and benchmark our approach against a well-established exploration method, we compare our results with DEP-RL. To ensure a comprehensive comparison, we evaluate three distinct seeds of the stochastic DEP-RL without curricula (NonCurr DEP), directly targeting all velocities from the running phase. The results, illustrated in Fig. 4a alongside the Schedule 2-4-8 results for comparison, show that DEP-RL achieves relatively low rewards, indicating ineffective task learning.

#### Evaluating robustness

**Fig. 5:**
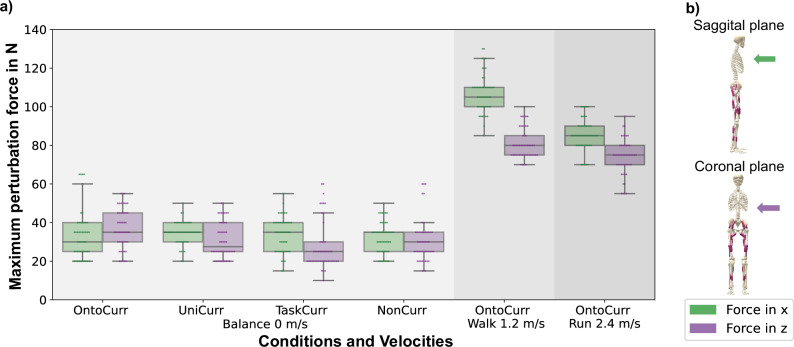
Robustness to perturbation forces. **a** Boxplots illustrate the maximum perturbation forces agents withstood when applied to the torso’s center of mass in the sagittal (*x*-direction) and coronal (*z*-direction) planes, as illustrated in the accompanying schematic. Data is presented for balance (0 ms-1), walking (1.2 ms-1), and running (2.4 ms-1), with five rollouts for each of the ten seeds (*n* = 50 per condition). The center line of each box indicates the median of the maximum perturbation forces withstood, the box limits correspond to the upper and lower quartiles, whiskers extend to 1.5 times the interquartile range, and points outside this range are considered outliers. We compare a baseline without curriculum (NonCurr), only the task curriculum (AdultCurr), the ontogenetic morphological and task curriculum (OntoCurr), and the uniform morphological and task curriculum (UniCurr). Due to substantial target velocity errors in other conditions, only OntoCurr is shown for walking and running speeds. **b** Schematic representation of the perturbation forces and their directions.

Despite DEP-RL and two curricula failing to learn the full set of tasks, OntoCurr not only learns different velocity gaits and balance but also demonstrates strong robustness. Figure 5 presents the maximal perturbation forces withstood in the coronal and sagittal planes for five rollouts of each of the ten seeds at various velocities: balance 0 ms-1, walking 1.2 ms-1 and running 2.4 ms-1, as in our target velocity analysis. In each experiment, perturbation forces occured every 4 s, each lasting 1 s. Perturbations alternated direction within each run, either along the x-axis (forward-backward within the sagittal plane) or the z-axis (left-right within the coronal plane). The initial direction was randomized at the start of each trial, and the alternating sequence continued until trial termination. At 0 ms-1, the perturbation forces necessary to destabilize the agent are compared across different conditions, as all target velocity errors were relatively low (Fig. 3c7). For forces applied along the x-axis, UniCurr, AdultCurr, and NonCurr conditions exhibit similar median forces of ~35 N. However, along the z-axis, AdultCurr shows the lowest median force, while OntoCurr demonstrates the highest. Although its median force along the x-axis is 5 N lower than other conditions, OntoCurr achieves the highest non-outlier maximum forces of 60 N along the x-axis and 55 N along the z-axis. For walking and running, only the OntoCurr condition is considered, as other strategies resulted in higher target velocity errors (Fig. 3c8, c9), making direct comparisons less valid. For walking, the agent withstands the highest perturbation forces, with maximum values reaching 130 N along the x-axis and 100 N along the z-axis. The maximum forces the agent withstands while running are 100 N along the x-axis and 95 N along the z-axis, indicative of strong stability under higher velocities.

Despite being trained at a single velocity per episode, the agent’s ability to adapt to gradually and abruptly changing velocities within an episode further underscores its flexibility and adaptability to dynamic environments. This robustness is highlighted by the agent’s success in navigating various challenging terrains, including gaps, stairs, and hurdles, even though training was conducted exclusively on flat terrain. The videos on our website (https://sites.google.com/view/bioinspired-double-curriculum/home) illustrate this robust behavior in action. For the velocity increase condition, the target velocity starts at 0 ms-1 for 100 steps, gradually increases from 1.2 ms-1 to 3.4 ms-1 within 700 steps, and finally stays at 3.4 ms-1 for 200 steps. The velocity decrease follows the reverse pattern: starting at 3.4 ms-1 for 100 steps, gradually slowing to 1.2 ms-1 over 700 steps, and then abruptly droping to 0 ms-1 for 200 steps. These protocols were chosen to challenge the agent with varying velocity changes, thereby assessing the robustness and flexibility of the learned control strategies.

## Discussion

Our bioinspired double curriculum framework, combining morphology and task curricula, successfully addresses RL exploration challenges in musculoskeletal systems due to intricate dynamics and over-actuation, demonstrating state-of-the-art performance. This combined approach enables the agent to efficiently learn robust gaits and upright standing that are adaptable to varying velocities and perturbations, within a unified policy.

Training NonCurr and AdultCurr results in the agent acquiring only upright standing. Interestingly, the AdultCurr achieves the highest reward during the balance phase, likely due to the adult morphology’s natural preference for more stationary behavior with less exploration, as indicated by its lower positional and velocity coverage in comparison to the other morphologies (Fig. 2).

In contrast, acquiring a broader range of velocities required the combination of both morphological and task curricula, as seen in OntoCurr. The Onto4y morphology, in particular, exhibits the most superior space and velocity coverage (Fig. 2), facilitated by its lightest legs which in term have a lower rotational inertia. Additionally, the lighter legs play a crucial role in stabilizing the heavy torso, where excessive rotation could induce instability and falls. As such, the enhanced dynamical behavior of the morphology is leveraged to learn diverse locomotion behaviors with the Onto4y before transitioning to the adult morphology to continue mastering all gaits and balance.

An alternative approach, UniCurr with the Uni4y model, where all body segments are uniformly scaled down, underscores the importance of scheduling in curriculum learning. Initially unsuccessful, UniCurr achieved learning with some seeds under the extended Schedule 3-6-12 and all seeds using Schedule 4-8-16 (Fig. 4b-c) which allocated more environment steps to balance, walking and running phases with Uni4y. Compared to OntoCurr, UniCurr required more environment steps with the 4 year-old morphology due to its heavier legs, which reduced position and velocity coverage, limiting exploration. Furthermore, incorporating a 12 year-old morphology in an intermediate Schedule 2−4−8−12 (Fig. 4d) did not improve rewards. Since Uni12y’s exploration capacity falls between Uni4y and the adult morphology, its uniform scaling and heavier leg mass were insufficient to drive learning using this schedule. Finding the optimal schedule is an ongoing empirical challenge^24,36^. Our work shows that the optimal schedule for each phase depends on the morphology’s exploratory characteristics. While we explored several curricula schedules and demonstrated the generality of our findings (Fig. 4), future work could explore automated approaches to adapt schedules dynamically. Existing scheduling strategies such as reward-based curriculum learning^5^, particle filtering methods^25^, and value function-based approaches^37^ could present promising directions to further enhance the efficiency of our framework.

Our bioinspired double curriculum approach outperforms DEP-RL by enforcing structured exploration. DEP-RL, which enforces strongly correlated muscle activations, has proven effective in muscle-actuated systems but failed to solve our task (Fig. 4a). Its broad, unstructured exploration proved ineffective compared to our curricula, which offer two advantages: Importantly, guided optimization, where the enhanced dynamics of the 4 year-old morphology initially improve exploration guiding the learning process, and secondarily structured learning, where combining morphological and task curricula dynamically reshapes the solution space throughout training, preventing premature convergence. These findings align with prior work showing that structured morphological evolution guides exploration more effectively than noise-based methods^38^. Beyond performance gains, our approach seems to reduce computational complexity by shifting the exploration burden from the controller to the morphology. Unlike DEP-RL and other methods requiring complex exploration strategies^11,17,19^, multi-head^39^ or recurrent architectures^6^, our bioinspired double curriculum enables efficient learning with a simple architecture, reducing computational overhead without sacrificing performance.

Our approach effectively generalizes to various robustness tests, both quantitatively with perturbation forces and qualitatively with varying terrain and changing velocities— none of which were encountered during training. At balance, maximum perturbation forces withstood across different conditions appear similar, likely due to higher velocity errors in NonCurr and AdultCurr conditions (Fig. 3c7). These errors indicate deviations from the upright position, making direct force comparisons unfair. Given the larger velocity errors observed at higher speeds, we focus our robustness assessment on OntoCurr, which closely follows the target velocities. This agent withstands perturbation forces of up to 130 N. While our agent’s robustness may not fully match human levels, especially in balance, this could be attributed to the sparse reward structure, strictly enforcing upright standing. Balancing, which requires the agent to remain stationary, is an outlier compared to the remaining tasks that allow for body segment movement. To improve robustness, considering partial rewards for minor deviations from the upright position could be beneficial. Nevertheless, our results demonstrate the agent’s ability to achieve robust and adaptable movement, providing a solid foundation for future enhancements.

**Fig. 6:**
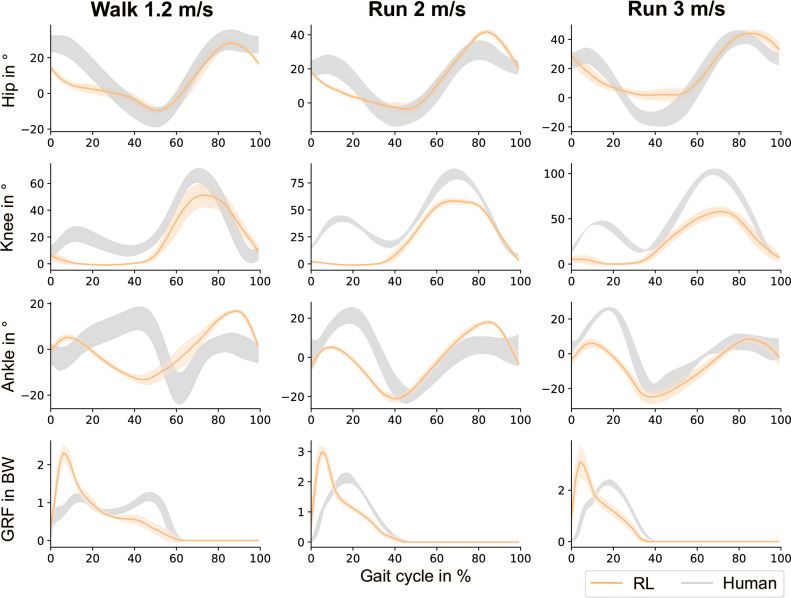
Bioinspired double curriculum produces gaits with good alignment to human patterns. Displayed are the hip, knee, ankle joint angles and ground reaction forces (GRF) during locomotion on flat ground at three different velocities. The means and standard deviations (orange), taken over all steps within a 10 s window from three rollouts across three different seeds, is compared to experimental data (gray) from walking^40^ and running at various speeds^41^.

To complement our analysis, we compare gait kinematics and ground reaction forces (GRF) with experimental data for walking^40^ and running at different speeds^41^. Results are shown in Fig. 6 for three rollouts from the three seeds exhibiting the most human-like policy, averaged over all gait cycles of both legs over 10 s. Our approach produced periodic gaits with good symmetry and alignment to human patterns. While some discrepancies were observed for walking, particularly in ankle angle and knee flexion, overall there was a strong resemblance to human patterns. This is especially noteworthy considering the challenges of learning natural low-speed walking, often leading to varied unnatural gestures with similar performance rewards^17^. As velocities increase, the ankle angles become more human-like in timing, while hip and knee flexion decrease. Knee flexion remains single-peaked for walking and running, lacking the early stance flexion typically seen in human gaits. The walking GRF profile exhibits a strong initial impact peak followed by a gradual decline, rather than the characteristic double-peaked pattern observed in human data. In contrast, the running GRF profiles maintain a single peak, but this peak occurs earlier than in experimental data and rises more steeply. These differences can be attributed to three main factors.

First, our musculoskeletal models incorporate morphological simplifications, arising from both contact mechanics and structural properties. These factors collectively contribute to the observed discrepancies in gait characteristics. The Hunt-Crossley contact model, which governs foot-ground interactions, relies on three contact points–one at the heel and two at the forefoot. With parameters inherited from the HyfyDy framework, it employs high contact stiffness (11006.4 Nm-1) and low damping (1 Nm-1), resulting in a highly elastic response upon impact. This leads to a sharper and higher initial peak in the GRF at touch-down and contributes to discrepancies in ankle kinematics. Additionally, the model’s rigid foot structure, which lacks a toe joint, further amplifies these discrepancies. In human walking, toe flexion and midfoot compliance allow for a gradual transfer of load during stance, enabling the characteristic double-peaked GRF. The absence leads to a premature unloading after mid-stance, eliminating a distinct push-off phase and resulting in a single-peaked GRF profile. Another key factor influencing gait dynamics is the model’s knee behavior. The stiff knee pattern observed during early stance may be attributed to the absence of joint limit sensors–crucial for human motor control. As a result, the agent naturally compensates by reducing knee flexion at first contact, making balance control easier. This could be avoided in the future by introducing a penalty for joint limit torques^20^. Since knee flexion dynamics influence GRF shape, this constraint directly contributes to the lack of a double-peaked GRF. Overall, the simplified anatomical structure of our model further affects gait dynamics, as the model includes a limited number of muscles, segments, and DOF. Specifically, the upper body is represented as a single rigid segment without arms or compliant structures, which likely exacerbates impact forces at touch-down and influences overall kinematics. Refining the model parameters and incorporating more anatomical detail might improve overall gait dynamics by reinforcing the morphological intelligence, however, our findings already exhibit symmetric, cyclic gaits with encouraging similarities to human locomotion.

Second, beyond morphological constraints, human locomotion is also shaped by optimality principles. Biological gait prioritizes energy efficiency, which not only ensures the lowest cost of transport at optimal walking speeds but also drives the transition between walking and running^42,43^. Previous approaches have relied on intricate neuromechanically-inspired reward structures to capture natural gait behaviors^20,22^, including terms for symmetry, pain, and energy efficiency. Integrating only the main optimality principles could make our results even more natural, particularly influencing knee kinematics and GRF characteristics. This refinement could make our approach more applicable to assistive devices and other future applications. However, our approach has already enabled the emergence of sophisticated locomotion skills—achieving a level of proficiency often thought to require detailed reward design— without any reward tuning.

Third, our approach relies on two key morphological stages, the 4 year-old and adult models, inspired by human growth. While this choice allowed us to focus on efficiently acquiring robust locomotion, incorporating additional stages could improve the naturalism of learned behaviors by mimicking lifelong motor learning.

These specific morphologies are not the only viable options for successful curriculum design. As, the relationship between morphology, environmental complexity, and learnability remains an open question^44–46^, finding the optimal morphologies is still a challenge. Optimizing embodied agents’ morphologies throughout training faces two key challenges: the vast search space of potential morphologies and the computational cost of evaluating their fitness^44,47^. To address this complexity, we take inspiration from nature as a principle starting point to simplify curriculum design while maintaining computational efficiency.

Exploring the effects of dynamic morphological changes beyond biological norms, we introduced OntoRand and UniRand, where morphology switched randomly during training, first without a task curriculum and later with both the original and expanded schedules (Fig. 4e and Supplementary Fig. 2a-b). These strategies demonstrate low, spiky rewards at the end of training, failing to learn all gaits. While randomization strategies may enhance generalization if a policy is successfully learned^48,49^, they do not provide the structured development of exploration necessary for solving our task. These results reinforce our nature-inspired approach, demonstrating the benefits of bioinspired schedules for effective learning.

To sum up, our bioinspired double curriculum approach is independent of the task, musculoskeletal model, and algorithm, suggesting versatility. By leveraging embodied intelligence, it eliminates the need for complex algorithms, intricate exploration techniques, imitation learning demonstrations, or extensive reward design. This enables efficient learning of close-to-natural, robust gaits and upright standing, providing a solid foundation for streamlined and adaptable solutions.

## Methods

### Preliminaries

A Markov Decision Process is defined as a tuple $({{\mathcal{S}}},{{\mathcal{A}}},P,r,\gamma ,{\mu }_{0})$, where ${{\mathcal{S}}}$ is the state space, ${{\mathcal{A}}}$ is the action space, $P:{{\mathcal{S}}}\times {{\mathcal{A}}}\times {{\mathcal{S}}}\to {{\mathbb{R}}}^{+}$ is the transition kernel that gives the probability of transitioning from state *s* to state $s{\prime}$ given action *a*, $r:{{\mathcal{S}}}\times {{\mathcal{A}}}\to {\mathbb{R}}$ is the reward function providing the reward received when taking action *a* in state *s*, *γ* is the discount factor, and ${\mu }_{0}:{{\mathcal{S}}}\to {{\mathbb{R}}}^{+}$ is the initial state distribution. At each step, the agent observes a state $s\in {{\mathcal{S}}}$ from the environment, selects an action $a\in {{\mathcal{A}}}$ according to the policy $\pi :{{\mathcal{S}}}\times {{\mathcal{A}}}\to {{\mathbb{R}}}^{+}$, and transitions to the next state $s{\prime} \in {{\mathcal{S}}}$ with probability $P(s{\prime} | s,a)$, receiving a reward *r*(*s*, *a*). In this work, we use parametric policies *π*(*a*∣*s*, ***θ***), where the policy is parameterized by ***θ*** and assumed to follow a specific probability distribution. Together with the transition kernel and the initial state distribution, this defines the stationary state distribution *μ*_*π*_(*s*). Finally, we define the probability of a trajectory *τ*_*t*_ = {(*s*_*t*_, *a*_*t*_), …, (*s*_*T*_, *a*_*T*_)} of length *T* starting from an initial state *s*_*t*_ and action *a*_*t*_ and following a policy *π* thereafter as ${p}_{\pi }({\tau }_{t}| {s}_{t},{a}_{t})={\prod }_{k = t}^{T-1}\pi ({a}_{k}| {s}_{k})\,P({s}_{k+1}| {s}_{k},{a}_{k})$.

### Policy optimization

In this work, we utilize the MPO algorithm^34^, as it demonstrated robust policy learning in prior locomotion studies^19,20^. MPO extends the classical RL problem by incorporating a relative entropy constraint. The optimization problem is defined as

1 $$	\mathop{\max }_{q} {{\mathbb{E}}}_{{\mu }_{\pi }(s)}\left[{{\mathbb{E}}}_{q(a| s)}[{Q}^{q}(s,a)]\right]\\ 	\,{\mbox{s.t.}}\,\quad {{\mathbb{E}}}_{{\mu }_{\pi }(s)}\left[\,{\mbox{KL}}\,\left(q(a| s),\pi (a| s,{{{\boldsymbol{\theta }}}}_{i})\right)\right] < \epsilon ,$$

where *q*(*a*∣*s*) is an auxiliary distribution, KL denotes the Kullback-Leibler divergence, and *ϵ* is a hyperparameter. The action-value function *Q*^*q*^(*s*, *a*) under policy *q* is defined as

2 $${Q}^{q}(s,a)=r(s,a)+{{\mathbb{E}}}_{{p}_{q}({\tau }_{t}| {s}_{t} = s,{a}_{t} = a)}\left[{\sum }_{t\ge 1}^{\infty }{\gamma }^{t}{r}_{t}\right].$$

In line with the original formulation, we employ function approximation to estimate the action-value function, denoted as *Q*^*q*^(*s*, *a*, ***ϕ***), which is trained to minimize the Bellman error. Using Equation (2), the solution to Equation (1) is the following closed-form variational distribution

3 $$q(a\, | \, s)\propto \pi (a\, | \, s,{{\boldsymbol{\theta }}})\exp \left(\frac{Q(s,a,{{\boldsymbol{\phi }}})}{{\eta }^{* }}\right),$$

which is estimated via samples, and where η^*^ is obtained by minimizing the convex dual function. Given *q*, the optimization of the policy then reduces to a supervised learning problem, where the policy parameters are optimized according to the following maximum a-posteriori objective

4 $${\max }_{{{\boldsymbol{\theta }}}}{{\mathcal{J}}}(q,{{\boldsymbol{\theta }}})={\max }_{{{\boldsymbol{\theta }}}}{{\mathbb{E}}}_{{\mu }_{q}(s)}\left[{{\mathbb{E}}}_{q(a| s)}\left[\log \pi (a\, | \, s,{{\boldsymbol{\theta }}})\right]\right]+\log p({{\boldsymbol{\theta }}}),$$

where *p*(***θ***) is a prior on the policy parameters. We refer to Abdolmaleki et al.^34^ for further details on MPO. In this work, we use similar hyperparameters as in Schumacher et al.^20^ for MPO.

The buffer size is set to 1 × 10^6^, with a batch size of 256. Initial steps before batches are 1 × 10^6^, with 1000 steps between batches, for a total of 30 batches. The n-step return is 1. Training is performed with 20 parallel and 10 sequential processes. The neural network comprises two hidden layers of 256 units each, using ReLU activation. The learning rates are 3 × 10^−4^ for both the actor and critic, and 1 × 10^−2^ for the dual variables. We executed our training on an NVIDIA RTX A6000 GPU and 64 CPU cores. For 6 × 10^7^ steps training required about 13 h.

### Exploration with muscle-actuated models

While RL algorithms have progressed rapidly, much of the literature dealing with embodied systems is focused on robotic setups. In contrast to those systems, where usually one independent actuator per DOF is present, the humanoids in this study possess a large number of mono- and bi-articular muscle-tendon-units per DOF^20^. Recent studies^11,14,15,19^, have shown that this over-actuated regime can be problematic for RL algorithms, which is why a method based on DEP^18,19^ is employed to facilitate the exploration problem. DEP-RL uses an exploration technique based on the maximization of correlations between muscle length velocities:

5 $$\tau \dot{C}={\dot{l}}_{t}{\dot{l}}_{t-{{\Delta }}t}^{{{\rm{T}}}}-C,$$

where *C* is the DEP control matrix, $\dot{l}$ is the muscle fiber velocity and Δ*t* is a time delay. DEP-RL was shown to improve exploration for RL methods in bipedal walking and running as well as reaching tasks^19,20^.

### Environment

The algorithm and the curricula are evaluated in SCONE simulator with the integrated 3D HyFyDy model H1622^50,51^. This model (Fig. 3) includes a head-torso segment, a pelvis, and three segments for each leg: thigh, shank, and foot. Each leg is powered by eleven muscles controlling the hip, knee, and ankle DOF. Each foot has three spherical contact points: two for the forefoot and one for the heel. Contact forces are calculated using the Hunt-Crossley model^52^ with a friction cone characterized by static, dynamic and viscous friction^53^.

For the morphological curricula, this adult model is downscaled to represent 4 year-olds and 12 year-olds. The age of 4 aligns with the fundamental movement phase in Gallahue’s hourglass model^3^, where children develop motor control and rhythmic coordination of basic skills. Following Sutherland’s hypothesis^28^, changes in walking after the age of 4 are primarily due to limb length rather than central nervous system development, simplifying the scaling process. The age of 12 is chosen to represent pre-growth spurts, which typically peak at the age of 14 in boys^29^, where bone mass increases without proportional muscle strength growth, potentially complicating scaling^54^.

Overall, the main differences between the ontogenetically and uniformly scaled models are in their segment lengths and mass distribution, although both models maintain the same overall mass. For the ontogenetic models, each body segment is scaled down using anthropometric data from Snyder et al.^55^, with the mass distribution adjusted to fit the revised body configuration. This results, especially for Onto4y, in proportionally larger torsos and smaller pelvises, femurs, and tibias compared to the adult model. In contrast, the uniformly scaled model applies a common ratio based on total body height across all segments, maintaining the adult model’s mass distribution.

Complementing these scaling adjustments, we modify other key parameters in the model. Muscle parameters are modified to ensure a consistent moment-angle relationship across different age groups during maximum muscle activation, following O’Brien’s^56^ observations during maximum voluntary contraction. Muscle length parameters are adjusted according to the previously described length scaling ratios. Maximal muscle force and stiffness parameters of both ground contact points and joints are scaled according to the corresponding mass-length scaling law as described by Correa et al.^57^ and Geyer et al.^58^, respectively. For stiffness, this adjustment ensures that dynamic locomotion remains invariant with size changes, maintaining constant normalized stiffness–and by extension, absolute stiffness–throughout the models^58^. More information on the skeletal and muscle parameters can be found in Supplementary Materials 2 including Supplementary Table 1–3. The detailed outline of the models and scaling procedure follows the method described by Badie and Schmitt^23^.

To inform the learning algorithm about the environment’s state at each iteration, the agent receives the current observation state at 100 Hz, encompassing diverse environmental features alongside information about the target velocity and position. We limit the horizon of an episode to 1000 steps corresponding to 10 s and include the following information in the environmental features: muscle length, velocity and force, torso orientation and angular velocity arrays, angles and angular velocities for all 19 DOF and vertical GRF on the right and left. These features are inspired by the sensor fusion at multiple levels in humans, including golgi tendon organs, muscle spindles, skin and joint mechanoreceptors, and vestibular receptors^59^. We complement these observations with task-specific information: the difference between actual and target velocity along the x- and y-axes and the difference between actual and target position along the z-axis—for stronger movement control in comparison to velocity. Though introducing the target velocity adds some noise during switching, it provides valuable feedback to the controller. To ensure consistency across models, all differing observations are scaled appropriately. Muscle information is normalized, while other values are adjusted using dimensionless numbers derived from gait mechanics, following Hof’s framework^60^: force $\hat{F}=\frac{F}{mg}$, length $\hat{l}=\frac{l}{{l}_{0}}$, velocity $\hat{v}=\frac{v}{\sqrt{g{l}_{0}}}$, and angular velocity $\hat{\omega }=\frac{\omega }{\sqrt{\frac{g}{{l}_{0}}}}$ with g gravity, m body mass and *l*_0_ leg length.

### Reward

To teach the environments, depending on the task, a different reward function is employed. We distinguish between learning to balance and learning gaits based on the target velocity. The reward function for balance includes four performance measures: forward velocity along x-axis ${R}_{{v}_{x,{{\rm{b}}}}}$, vertical velocity along y-axis ${R}_{{v}_{y,{{\rm{b}}}}}$, lateral position along z-axis ${R}_{{p}_{z,{{\rm{b}}}}}$, and torso orientation around z-axis ${R}_{{o}_{z,{{\rm{b}}}}}$. The reward function for walking and running consists of two performance measures: forward velocity ${R}_{{v}_{x},{{\rm{g}}}}$ and lateral position *R*_*z*,g_. The total reward function comes together as

$$R=\left\{\begin{array}{ll}{R}_{{v}_{x,{{\rm{b}}}}}+{R}_{{v}_{y,{{\rm{b}}}}}+{R}_{{p}_{z,{{\rm{b}}}}}+{R}_{{o}_{z,{{\rm{b}}}}},\quad &\,{\mbox{if}}\,{v}_{x}^{* }=0\\ {c}_{{v}_{x,{{\rm{g}}}}}{R}_{{v}_{x,{{\rm{g}}}}}+{c}_{{p}_{z,{{\rm{g}}}}}{R}_{{p}_{z,{{\rm{g}}}}},\hfill\quad &\,{\mbox{otherwise}}.\,\end{array}\right.$$

All performance measures follow the same structure based on an exponential function as the equations used in^20^

6 $$R={{{\rm{e}}}}^{-k{\left(\frac{{a}^{* }-a}{s}\right)}^{2}}.$$

Here, *a* represents the variable being rewarded, a^*^ denotes the desired value, *k* controls the reward’s sensitivity to deviations from the target, and *s* serves as the scaling factor based on Hof’s scaling laws^60^, as described above. This scaling ensures fair rewards across different musculoskeletal models. Additionally, the target velocities described above are scaled to the specific models, ensuring that smaller models learn movements at appropriate velocities, preserving dynamic similarity^33^. We prioritize forward reward by setting ${c}_{{v}_{x,{{\rm{g}}}}}$ to 3.5 over lateral reward with ${c}_{{p}_{z,{{\rm{g}}}}}$ at 0.5 during non-balance runs, ensuring the total maximal reward remains consistent with that of the balance task. The parameters ${k}_{{v}_{x,{{\rm{b}}}}}$, ${k}_{{v}_{y,{{\rm{b}}}}}$, ${k}_{{p}_{z,{{\rm{b}}}}}$, and ${k}_{{o}_{z,{{\rm{b}}}}}$ are all assigned a value of 500, while ${k}_{{p}_{z,{{\rm{g}}}}}$ is set to 10. Additionally, the parameter ${k}_{{v}_{x,{{\rm{g}}}}}$ is defined as $\frac{10\sqrt{l}}{{v}_{x}^{* }}$, where *l* represents the leg length ratio of the different musculoskeletal models relative to the adult for fair reward, and ${v}_{x}^{* }$ is the desired velocity. This formulation encourages higher velocities while preventing instability and poor performance caused by large deviations between target and actual velocities.

## Supplementary information

Supplementary Information

## Data Availability

All data that support the findings of this study are available in DaRUS with the identifier 10.18419/DARUS-5030. Furthermore, we provide videos on our website that showcase the robustness of the learned gaits.

## Code availability

The code used to generate the results of this study is included in DaRUS with the identifier 10.18419/DARUS-5030.
