# Supplementary material for: Bioinspired morphology and task curricula for learning locomotion in bipedal muscle-actuated systems
Source: Commun Eng. 2025 Jun 20;4:115. doi: 10.1038/s44172-025-00443-0 (PMC12181307; doi:10.1038/s44172-025-00443-0)
Supplement: Supplementary file 1 — Supplementary Information [file 44172_2025_443_MOESM1_ESM.pdf]

Bioinspired morphology and task curricula for learning locomotion in bipedal muscle-actuated systems

Supplementary Materials

Nadine Badie[1], Firas Al-Hafez[2], Pierre Schumacher[3,4], Daniel F. B. Haeufle[4,5], Jan Peters[2], Syn Schmitt[1,5]

[1] Institute for Modelling and Simulation of Biomechanical Systems, University of Stuttgart, Nobelstraße 15, Stuttgart, 70569, Germany
[2] Institute for Intelligent Autonomous Systems, TU Darmstadt, Hochschulstraße 10, Darmstadt, 64289, Germany
[3] Max Planck Institute for Intelligent Systems, Max-Planck-Ring 4, Tübingen, 72076, Germany
[4] Hertie-Institute for Clinical Brain Research, University of Tübingen, Otfried-Müller-Str. 27, Tübingen, 72076, Germany
[5] Center for Bionic Intelligence Tübingen-Stuttgart (BITS), Universities of Stuttgart and Tübingen, Nobelstraße 15, Stuttgart, 70569, Germany

1 Supplementary Results

1.1 Morphological curriculum

To assess whether the increased exploration in younger morphologies is a robust effect that persists in systems with higher degrees of over-actuation, we conducted an additional experiment. Specifically, we expanded the action space by applying a multiplier n as proposed by Schumacher et al.[1]. To approximate the number of muscles in the human body[2] and achieve a human-like level of over-actuation, we scale the control input with $n = 67$, resulting in 603 actions. The modified control vector was then averaged back to the original dimensions. Figure 1 displays the resulting position and velocity coverage range for all morphologies. Onto4y demonstrates the highest level of exploration, followed by Uni4y and the adult model. Thus, our toy experiment in an over-actuated setting consistently demonstrated Onto4y's superior exploration capabilities, suggesting OntoCurr's potential for handling more complex over-actuated systems.

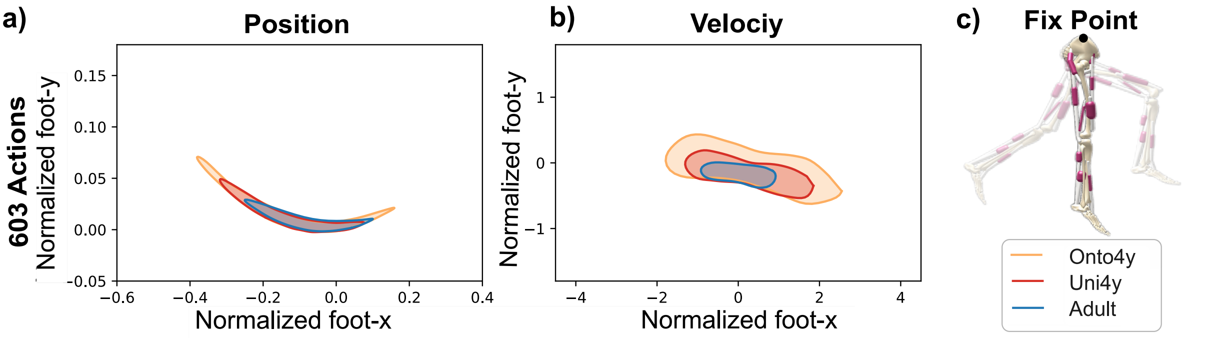

Figure 1: **Younger morphologies exhibit superior exploration, demonstrated by a broader kinematic workspace with increased positional and velocity coverage.** Over 50 episodes of 1000 steps ($\Delta t = 10\ s$), we tracked (a) foot trajectories and (b) velocities during pure exploration. The data for the ontogenetically scaled (Onto4y) and the uniformly scaled (Uni4y) 4-year-olds and the adult is presented in a dimensionless form, normalized according to the typical biomechanical approach of Hof[3], for the expanded action space of $a \in \mathbb{R}^{603}$. (c) Schematic illustrating the experimental setup, where the pelvis of each model was fixed in place while one leg was subjected to white noise perturbations.

1.2 Schedule ablation

We extend the non-bioinspired experiments by combining the random morphology schedule, where the agent alternates between the 4-year-old and adult morphologies throughout the entire training process, with the task curriculum. We evaluate two different schedules: Schedule 2-4 and Schedule 4-8. In Schedule 2-4, the agent starts with the balance phase and transitions to the walking phase at 2×10^6 environment steps, followed by the running phase at 4×10^6 steps. In Schedule 4-8, the balance phase extends to 4×10^6 steps, with walking continuing until 8×10^6 steps, before finally transitioning to the running phase.
The mean and standard deviation of rewards for three seeds are shown in Figure 2, where task transitions are shown by vertical dotted lines. During the balance phase, rewards for both OntoRand and UniRand rise, but as the following phase begins, they drastically decrease—roughly halving. During the running phase, rewards remain spiky and low.

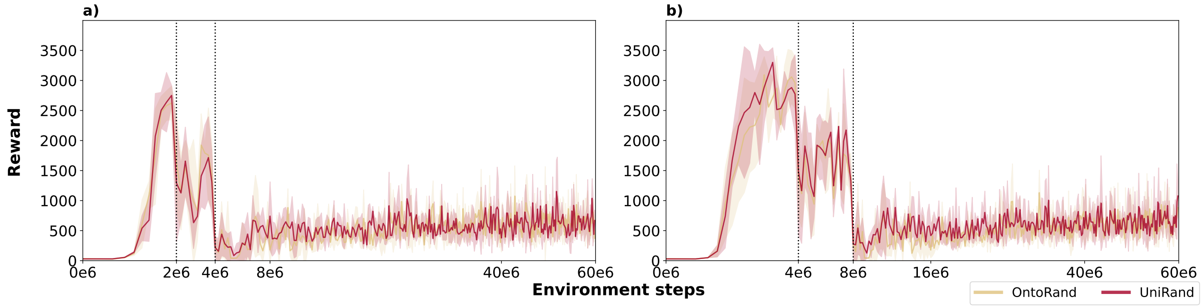

Figure 2: **Ablation study on random morphology combined with task curriculum.** The agent alternates continuously between morphologies in two settings: ontogenetically scaled 4-year-old and adult (OntoRand), and uniformly scaled 4-year-old and adult (UniRand). Both settings follow two task curriculum schedules, with dotted vertical lines indicating phase transitions: (a) Schedule 2-4 and (b) Schedule 4-8. Rewards and standard deviations are shown for three different seeds.

2 Supplementary Methods

Table 1: Masses for the ontogenetically scaled (Onto4y) and uniformly scaled (Uni4y) 4-year-olds, and the adult morphologies.

Segment	Mass in kg		
	Onto4y	Uni4y	Adult
Torso	10.639	7.258	34.237
Pelvis	1.682	2.497	11.777
Thigh	1.087	1.972	9.301
Shank	0.457	0.786	3.708
Foot	0.195	0.265	1.250

Table 2: Segment lengths ratios for the ontogenetically scaled (Onto4y) and the uniformly scaled (Uni4y) 4-year-olds using the integrated HyFyDy model H1622[4].

Segment	Length ratio	
	Onto4y	Uni4y
Torso	0.693	0.569
Pelvis	0.535	0.569
Thigh	0.500	0.569
Shank	0.509	0.569
Foot	0.551	0.569

Table 3: Muscle properties for the ontogenetically scaled (Onto4y) and uniformly scaled (Uni4y) 4-year-olds, as well as the adult morphology, including maximum isometric force, optimal fiber length, and tendon slack length.

Muscle	Maximum isometric force in N			Optimal fiber length in m			Tendon slack length in m		
	Onto4y	Uni4y	Adult	Onto4y	Uni4y	Adult	Onto4y	Uni4y	Adult
Gluteus Medius	828	765	2045	0.039	0.042	0.073	0.035	0.038	0.066
Adductor Magnus	958	849	2268	0.044	0.050	0.087	0.030	0.034	0.060
Hamstrings	1110	971	2594	0.049	0.056	0.098	0.159	0.182	0.319
Biceps Femoris	342	301	804	0.055	0.063	0.110	0.048	0.054	0.095
Gluteus Maximus	799	727	1944	0.081	0.089	0.157	0.058	0.063	0.111
Iliopsoas	891	818	2186	0.056	0.061	0.107	0.079	0.087	0.152
Rectus Femoris	496	437	1169	0.038	0.043	0.076	0.173	0.197	0.345
Vasti	1937	1695	4530	0.050	0.057	0.099	0.061	0.070	0.123
Gastrocnemius	937	839	2241	0.026	0.029	0.051	0.196	0.219	0.384
Soleus	1479	1328	3549	0.023	0.025	0.044	0.127	0.141	0.248
Tibialis Anterior	651	591	1579	0.035	0.039	0.068	0.126	0.138	0.243

References

[1] Pierre Schumacher, Daniel Häufle, Dieter Büchler, Syn Schmitt, and Georg Martius. Dep-rl: Embodied exploration for reinforcement learning in overactuated and musculoskeletal systems. *Preprint at https://arxiv.org/abs/2206.00484*, 2022.

[2] Seunghwan Lee, Moonseok Park, Kyoungmin Lee, and Jehee Lee. Scalable muscle-actuated human simulation and control. *ACM Trans. Graph.*, 38(4):1–13, 2019.

[3] At L Hof. Scaling gait data to body size. *Gait Posture*, 3(4):222–223, 1996.

[4] Thomas Geijtenbeek. Scone: Open source software for predictive simulation of biological motion. *J. Open Source Softw.*, 4(38):1421, 2019.
